# NSAID-Avoidance Education in Community Pharmacies for Patients at High Risk for Acute Kidney Injury, Upstate New York, 2011

**DOI:** 10.5888/pcd11.140298

**Published:** 2014-12-18

**Authors:** Soo Min Jang, Jennifer Cerulli, Darren W. Grabe, Chester Fox, Joseph A. Vassalotti, Alexander J. Prokopienko, Amy Barton Pai

**Affiliations:** Author Affiliations: Soo Min Jang, Darren Grabe, Albany College of Pharmacy and Health Sciences, Albany, New York, and ANephRx Albany Nephrology Pharmacy Group, Albany, New York; Jennifer Cerulli, Alexander J. Prokopienko, Albany College of Pharmacy and Health Sciences, Albany, New York; Chester Fox, University of Buffalo School of Medicine and Biomedical Sciences, Buffalo, New York; Joseph A. Vassalotti, Icahn School of Medicine at Mount Sinai, New York, New York. Soo Min Jan, Darren Grabe, Chester Fox, and Joseph Vassalotti are also members of the New York State Chronic Kidney Disease Coalition, Albany, New York.

## Abstract

**Introduction:**

Nonsteroidal anti-inflammatory drugs (NSAIDs) are frequently associated with community-acquired acute kidney injury (AKI), a strong risk factor for development and progression of chronic kidney disease. Using access to prescription medication profiles, pharmacists can identify patients at high risk for NSAID-induced AKI. The primary objective of this analysis was to evaluate the effectiveness of a community pharmacy–based patient education program on patient knowledge of NSAID-associated renal safety concerns.

**Methods:**

Patients receiving prescription medications for hypertension or diabetes mellitus were invited to participate in an educational program on the risks of NSAID use. A patient knowledge questionnaire (PKQ) consisting of 5 questions scored from 1 to 5 was completed before and after the intervention. Information was collected on age, race, sex, and frequency of NSAID use.

**Results:**

A total of 152 participants (60% women) completed both the pre- and post-intervention questionnaire; average age was 54.6 (standard deviation [SD], 17.5). Mean pre-intervention PKQ score was 3.3 (SD, 1.4), and post-intervention score was 4.6 (SD, 0.9) (*P* = .002). Participants rated program usefulness (1 = not useful to 5 = extremely useful) as 4.2 (SD, 1.0). In addition, 48% reported current NSAID use and 67% reported that the program encouraged them to limit their use.

**Conclusion:**

NSAID use was common among patients at high risk for AKI. A brief educational intervention in a community pharmacy improved patient knowledge on NSAID-associated risks. Pharmacists practicing in the community can partner with primary care providers in the medical home model to educate patients at risk for AKI.

## Introduction

More than 98 million nonsteroidal anti-inflammatory drug (NSAID) prescriptions were filled in 2012 (1). NSAIDs have accounted for more than 70 million prescriptions and 30 billion over-the-counter purchases (2). NSAIDs are also among the most common medications prescribed inappropriately to older Americans (1,3). Among a cohort of 12,065 participants in the cross-sectional National Health and Nutrition Examination Survey who had an estimated glomerular filtration rate (eGFR) between 15 and 50 mL/min/1.73m^2^, 5% reported using over-the-counter NSAIDs regularly and 66.1% had used these agents for 1 year or longer (4).

Frequent, unmonitored use of NSAIDs among high-risk patients is associated with the development of acute and chronic kidney injury (5). NSAID use is a common inciting factor for community-acquired acute kidney injury (AKI) (6). NSAID-induced AKI abruptly alters renal hemodynamics, lowering effective perfusion of the glomerulus (7,8).

Interruption of this regulatory pathway increases the risk for hemodynamically mediated AKI, especially in patients who depend on vasodilatory prostaglandins to maintain kidney perfusion (7,8). Concomitant use of antihypertensive drugs and NSAIDs has been associated with a 5-fold increase in AKI risk (9). The relative risk for AKI among concurrent users of NSAIDs and diuretics is 3-fold higher than the risk among nonconcurrent users, likely because of decreased intravascular volume and renal perfusion (9). Angiotensin-converting enzyme inhibitors (ACEIs) dilate efferent arterioles and reduce glomerular capillary pressure, inhibiting the ability of the efferent arteriole to constrict when the renin–angiotensin–aldosterone system is activated or afferent arteriole vasodilatation is insufficient (7,10). Both current and recent use of ACEIs has been associated with as much as a 3-fold increase in the risk for AKI (9). Differences in pharmacologic selectivity and potential to cause intrarenal hemodynamic changes exist among NSAIDs; however, NSAID-induced AKI depends also on patient factors, which limits the ability to predict outcomes according to each NSAID (11,12).

The implications of an episode of AKI are relevant to chronic kidney disease (CKD). After an episode of AKI, kidney function is presumed to be fully recovered if serum creatinine levels return to baseline. However, recent data showed that up to 70% of elderly patients were predisposed to progression and development of de novo CKD within 2 years of an episode of AKI (13,14). NSAIDs are an important contributor to risk for AKI and a more rapid progression of CKD. In a cohort analysis of more than 10,000 patients aged 66 years or older, a high dose of NSAIDs was associated with a 26% increase in the risk for a decline in eGFR of more than 15 mL/min/1.73 m^2^ within 2 years (15).

This increased risk for adverse kidney events related to NSAIDs prompted the National Kidney Foundation to recommend displaying a clear warning on over-the-counter NSAID labels in 1985 (16). The NSAID Patient Safety Study collected data on NSAID use in primary care practices in Alabama (17). Patients who were identified as current NSAID users were contacted by telephone to participate in a survey. Among the survey participants, 63% used both over-the-counter and prescription NSAIDs, and only 13.7% patients recalled discussing NSAID use with a pharmacist. The authors concluded that pharmacists and pharmacy staff are missing an opportunity to provide counseling to high-risk patients to avoid inappropriate and unsafe NSAID use. The patient surveys indicated that a community pharmacy intervention could be valuable in increasing awareness of the risks of NSAID-induced AKI.

Provision of NSAID avoidance education to patients at risk for AKI is an important but underappreciated prevention strategy. Community pharmacists are readily accessible to these high-risk patients as they visit the pharmacy for prescription refills and over-the-counter purchases (18,19). The primary objective of this pilot project was to design and evaluate the effectiveness of a community pharmacy–based patient education program to increase awareness of the safety issues associated with NSAID use among patients at high risk for AKI. The primary outcome measure was patient knowledge questionnaire (PKQ) scores before and after the intervention. Secondary objectives were to quantify current use of NSAIDs and to determine whether the intervention encouraged patients to reduce their NSAID use.

## Methods

### Structured patient education intervention

Pharmacy interns (n = 24) in their last year of the doctor of pharmacy program at Albany College of Pharmacy and Health Sciences delivered the NSAID avoidance education program. The program was a requirement for interns who were completing a 6-week experiential rotation in a Community Pharmacy Advanced Pharmacy Practice Experience site during 3 consecutive 6-week rotations from January through April 2011 (a total of 18 weeks). The program was part of a larger educational initiative, the Wellness Targeted Intervention Program (18,19), which requires interns to deliver structured educational interventions during their community pharmacy rotations.

Pharmacy interns were educated on CKD and NSAID-induced hemodynamically mediated AKI by nephrology-trained doctors of pharmacy faculty (A.B.P. and D.W.G.) on the first day of rotation. The education module for interns and pharmacists was delivered in 90 minutes and included an interactive lecture-based format and self-assessment questions. The material on renal hemodynamics and the effects of medications, including NSAIDs, is covered topically in the intern’s core curriculum. As part of the rotation, the topic was reviewed more in depth, and the interns’ knowledge was assessed before delivery of the patient education program. The program provided to the interns and pharmacists included information on how the benefits of low-dose aspirin outweigh the risks of its use for primary or secondary prevention of cardiovascular disease. Because the primary purpose of the intervention was to increase awareness of the safety issues associated with NSAIDs, interns advised program participants to discuss NSAID use further with their primary care provider and offered counseling on topical agents and acetaminophen as alternatives under the direction of their supervising pharmacist. 

### Study population

Our target population for the education program was adults (≥18 y) who were at high risk for NSAID-induced AKI. We reviewed the prescription-dispensing–system medication profiles of adults receiving a new or refilled prescription in a convenience sample of 17 pharmacies (chain [n = 5], mass merchandiser [n = 4], supermarket [n = 5], independent [n = 3]) in the Community Pharmacy Advanced Pharmacy Practice Experience site network in the capital region of upstate New York. We identified potential participants as those taking antihypertensive medications (ACEIs, angiotensin-receptor blockers [ARBs], aliskiren, or diuretics), diabetes medications (oral or injectable), or digoxin. No patient identifiers were collected. Pre-intervention and post-intervention PKQs and prescription-dispensing–system medication profiles were numbered by investigators; these numbers were used as identifiers for pharmacy sites and participants. We excluded patients who declined to participate or were unable to read or understand English. Interns recruited participants during prescription pick-up or while patients were waiting for their prescriptions to be filled. Brightly colored notes placed on prescription bags of eligible patients alerted pharmacy staff and interns to engage those patients. Interns also recruited patients while conducting nondispensing activities, such as those during over-the-counter consultations, blood pressure clinics, or NSAID avoidance outreach programs. Patients who declined to participate were given a handout that covered the intervention’s key messages (Box 1).

**Box 1. Text of Patient Education Handout Given to 182 Patients in Community Pharmacies in the Capital Region of Upstate New York, 2011**

- Nonsteroidal anti-inflammatory drugs, or NSAIDs, are over-the-counter pain relievers. Common types of NSAIDs are ibuprofen (Motrin, Advil) and naproxen (Aleve). There are more examples on the back of this sheet.
- People with diabetes and high blood pressure are at risk for kidney disease.
- NSAIDs may not be good for people at risk for kidney disease because they may cause bad kidney effects — such as lowering blood supply to the kidney.
- Adding NSAIDs to some blood pressure medicines can increase the possibility of having bad kidney effects, including lack of good blood flow to the kidneys.
- People with high blood pressure and/or diabetes should avoid NSAIDs even short term and should always contact their pharmacist or other care provider before they consider using an NSAID.

Patients willing to participate in the program were asked to complete a baseline PKQ before engaging in the intervention. The intervention consisted of a review of the handout and an intern–participant discussion to address each educational objective. After the intervention, the participant completed the post-intervention PKQ and a short questionnaire. The average time for the intervention, including completion of both PKQs, was 10.3 minutes (SD, 3.5 min), according to completed documentation for 53 participant encounters. The interns collected information on medication history (from the patient and the prescription-dispensing–system medication profile). 

### Development of the PKQ

For the primary outcome measure, 5 patient-knowledge questions (Box 2) were developed by 2 nephrology-trained pharmacists (A.B.P. and D.W.G.) and an advanced community-practice pharmacist (J.C.). These questions were designed to test the knowledge of participants on 3 learning objectives: 1) The patient will be able to identify common NSAID products by generic/brand, 2) The patient will be able to understand the risks associated with NSAID use, and 3) The patient will be able to understand a course of action they should take to prevent adverse effects from NSAIDs.

**Box 2. Text of 5 Patient Knowledge Questions on NSAIDs Administered to 182 Patients in Community Pharmacies in the Capital Region of Upstate New York, 2011**

1. Which of the following medications is a nonsteroidal anti-inflammatory drug, or NSAID?
  - Acetaminophen (Tylenol)
  - Ibuprofen (Motrin)
  - Pseudoephedrine (Sudafed)
  - Menthol arthritis rubs (Mineral Ice, Icy Hot)
2. People at risk for kidney disease include people with which of the following medical conditions?
  - high blood pressure
  - diabetes
  - asthma
  - a and b only
3. NSAIDs may not be safe to use in patients with kidney disease or at risk for kidney disease.
  - True
  - False
4. Adding NSAIDs to prescription medications for high blood pressure can increase the risk of having bad kidney effects.
  - True
  - False
5. If someone has high blood pressure or diabetes, what should they do if they are in need of a pain reliever?
  - They should avoid NSAIDs even short term.
  - NSAIDs are not a problem in patients with high blood pressure or diabetes.
  - They should contact their pharmacist or other care provider before they consider using an NSAID.
  - a and c only

The questions were peer reviewed and piloted with 7 lay people for readability and clarity before being used in the study. Questions on age, race, and sex were included in the pre-intervention PKQ, and questions on current NSAID use and frequency of use were included in the post-intervention PKQ. Also, the post-intervention PKQ asked participants to rate the usefulness of the program on a 5-point Likert scale (1 = not useful to 5 = extremely useful) and to indicate whether the program encouraged them to avoid NSAID use in the future. If participants indicated the program did not encourage them to avoid NSAIDs, they were asked to explain why not in an open-ended response. The project was approved by the Albany College of Pharmacy and Health Sciences institutional review board.

### Analysis

We performed a descriptive analysis (mean, standard deviation [SD], range, or frequency) of all data. Only data on participants who completed both the pre-intervention and post-intervention PKQ were included in analysis. PKQs were scored according to the number of correct answers (range, 0 to 5). Pre-intervention and post-intervention PKQ scores were compared using a paired Student *t* test. Differences in characteristics (age, sex, and race) associated with poor health literacy among certain populations with kidney disease were compared using the unpaired Student *t *test (20). Comparisons of categorical data were analyzed by χ^2^ test. One-way analysis of variance (ANOVA) with Bonferroni correction was performed to evaluate percentage changes in pre-intervention and post-intervention PKQ scores by pharmacy type and pharmacy intern. All analysis was performed in STATA/SE 13.1 (StataCorp LP).

## Results

During the 18-week intervention, 182 patients participated in the education program, and 152 participants were included in the analysis. Of the 152 participants, 55 (36%) were recruited at supermarket pharmacies, 42 (28%) at chain pharmacies, 31 (20%) at mass merchandiser pharmacies, and 24 (16%) at independent pharmacies.

Average age was 54.6 (SD, 17.5); 76% were white; 60% were women (Table). The mean (SD) score on the pre-intervention question on avoidance was 3.3 (1.4), and the mean post-intervention score was 4.6 (0.9) (*P* < .001). The scores of white participants and black participants significantly increased after the intervention (Figure). Women had slightly lower scores than men before the intervention (women, 3.2 [1.5]; men, 3.4 [1.4], *P* = .35); however, both women and men had significantly higher scores after the intervention (women, 4.4 [0.9]; men, 4.7 [0.7]; *P* < .02 for pre–post comparison). Participants aged 65 or older had lower scores (2.9 [1.3]) than participants younger than 65 years (3.4 [1.5]) (*P* = .049). Participants who scored less than 100% on the post-intervention PKQ were older (mean age, 60 y [SD, 15 y]) than those who scored 100% (53 y [17 y]) *P* = .02). Although differences were not significant, those who scored less than 100% tended to be women (72% vs 48% of men) and had a lower rate of NSAID use (7% of participants who scored less than 100% vs 12% of participants who scored 100% reported current use of NSAIDs).

**Table:** Demographic Characteristics of Participants (N = 152) in an NSAID Avoidance Education Program at Community Pharmacies in the Capital Region of Upstate New York, 2011^a^

Characteristic	No. of Participants (%)
**Age^b^**	
Mean (SD), y	54.6 (17.5)
<65	105 (69)
≥65	43 (28)
**Sex^c^**	
Male	58 (38)
Female	91 (60)
**Race^d^**	
White	115 (76)
African American	23 (15)
Hispanic	5 (3)
Asian	1 (1)
American Indian	1(1)
**Current NSAID use^e^**	
Yes	73 (48)
No	68 (45)
Don’t know	5 (3)
**Frequency of NSAID use^f^**	
1 or 2 times per month	45 (53)
1 or 2 times per week	21 (25)
More than 1 or 2 times per week	19 (22)

Abbreviations: NSAID, nonsteroidal anti-inflammatory drug; SD, standard deviation.

a Values are no. (%) unless otherwise indicated.

b 4 participants did not respond.

c 3 participants did not respond.

d 7 participants did not respond.

e 6 participants did not respond.

f 11 participants reported a frequency of use although they also indicated they did not use NSAIDs; 2 participants reported a frequency although they also indicated they did not know whether they used NSAIDs, and 1 participant who reported NSAID use did not report a frequency.

**Figure:**
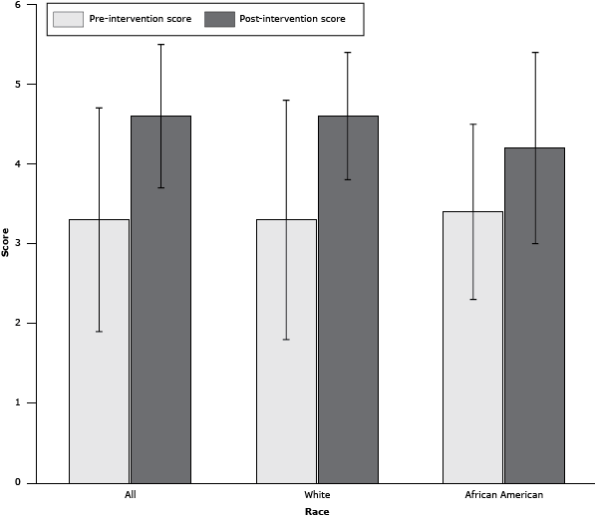
Mean pre-intervention and post-intervention scores on a patient knowledge questionnaire, by self-reported race, in the Capital Region of Upstate New York, 2011 (n = 145). Error bars indicate standard deviation. Scores differed significantly from pre- to post-intervention for all participants (n = 145) (*P* < .001), white participants (n = 122) (*P* = .001), and African American participants (n = 23) (*P* = .02). **Table:** 

Race	Pre-Intervention PKQ Score, Mean (SD)	Post-Intervention PKQ Score, Mean (SD)
All	3.3 (1.4)	4.6 (0.9)
White	3.3 (1.5)	4.6 (0.8)
African American	3.4 (1.1)	4.2 (1.2)

Abbreviations: PKQ, patient knowledge questionnaire; SD, standard deviation.

The use of ACEIs, ARBs, and diuretics was similar for participants who scored less than 100% and for those who scored 100% on the post-intervention PKQ. By type of pharmacy, participants at chain pharmacies showed the least improvement in score (*P* < .01 in comparison of chain pharmacies with all other types of pharmacy). By intern, we found no significant differences in score changes. Three-quarters of participants rated the intervention as very useful (n = 46) or extremely useful (n = 68); program usefulness was scored as 4.2 (SD, 1.0) overall.

Overall, 48% reported current NSAID use. Among NSAID users, 45% used NSAIDs 1 or 2 times per month, 29% used them 1 or 2 times per week, and 26% used them more than 1 or 2 times per week. The most common concomitant medications among participants with current prescription (n = 19) or over-the-counter NSAID use (n = 54) were ACEIs or ARBs (27%), oral antidiabetic agents (27%), β blockers (15%), and diuretics (15%). The combination of an ACEI or ARB with a diuretic was reported by 7% of NSAID users. Concomitant use of an ACEI with an ARB or an ARB with aliskiren, which is associated with labeled warnings and contraindications for participants with or at risk for kidney disease, was recorded for 2 participants. Sixty-seven percent reported that the program encouraged them to limit their use; 72% reported that this program encouraged them not to use NSAIDs for pain control. We found no significant correlation between pre-intervention PKQ score and perceived value of the program. 

## Discussion

Patient education about kidney disease and kidney-related risks is complex and requires multidisciplinary patient education interventions (21). An intensive education program focused on NSAID-induced gastrointestinal bleeding delivered by nurse practitioners in a primary care setting reduced dosage and overall use of prescription NSAIDs at 6 months among participants to a greater extent than did delivery of written materials (22); however, the study’s randomized design could have caused the Hawthorne effect, whereby study participants modified their behavior simply because they were being observed by study investigators. One drawback of primary care office–delivered education programs is the length of time between follow-up appointments. Our community-pharmacy–based intervention, in which counseling and education are initiated when prescriptions are picked up or over-the-counter NSAID purchases are made, allows for more frequent reinforcement of patient knowledge. This type of education program can complement and augment chronic disease education provided by primary care practitioners, promoting and improving the concept of the medical neighborhood for the patient-centered medical home (23).

Administration of a pre-intervention and post-intervention PKQ is a validated approach to assessing the effectiveness of an education program. Previous studies using this approach concluded that pre-intervention and post-intervention PKQs were useful to assess patients’ knowledge and were reliable, valid, and reproducible (24–26). Pharmacist-delivered education programs using PKQs successfully addressed unsafe use of other medications (27).

In our high-risk patient population, 48% reported current use of NSAIDs despite high risk for development of AKI. This rate is higher than some previously reported rates, perhaps because we collected data on over-the-counter use of NSAIDs and included patients who may have been at risk for CKD but did not have it (28). Given the extensive use of both prescription and over-the-counter NSAIDs and the prevalence of people at high risk for AKI in the U.S. population, awareness of the risk for AKI among health care professionals needs more attention. Robust data corroborate the idea that an episode of AKI can lead to development of CKD or accelerate the progression of existing CKD (13,29). The clinical practice guidelines of the organization Kidney Disease: Improving Global Outcomes state “all people with CKD are considered to be at increased risk for AKI” (30). In a nested-case–control study of nearly 400,000 patients aged 50 to 84 years, current NSAID use was associated with a 3-fold higher risk for AKI (8). The risk for AKI declined after NSAID treatment was documented to be discontinued by refill history. The impact of NSAID discontinuation on AKI underscores the importance of education on the appropriate use of NSAIDs. An analysis of 450 patients (35% of whom were 56 years or older) receiving NSAID prescriptions from a primary care center showed that only 14% of patients had their kidney function determined before initiating therapy. This study illustrates that risk for CKD and AKI is not routinely assessed among those for whom NSAIDs are prescribed.

Health literacy is an important consideration in patient education programs and success of educational interventions. We found that older participants had lower baseline PKQ scores than younger participants. This finding is consistent with research that identified older age as a risk factor for low health literacy (20). Our data can guide future education programs to focus on older patients, who are at especially high risk for AKI.

Success of any patient education program is due in part to perception of the intervention among participants. Importantly, 75% of participants in our program found it “very useful” or “extremely useful.” Community-based pharmacy education programs have been shown to be successful and sustainable with other targeted intervention programs (18,19,31,32).

This study has several limitations. Retention and application of knowledge over time was not assessed after the post-intervention PKQ was administered; the lack of a second post-intervention PKQ is one limitation. Performing any test of knowledge immediately after training does not indicate how long the individual will retain the information (26). In addition, this study used a convenience sample drawn from largely affluent suburban communities and thus limits the applicability to other populations. Deficits in health information technology and pharmacy-dispensing software interfaces limited access to comorbidity data. Although the intervention was generally well accepted by participants, 30 participants did not complete the post-intervention PKQ. Others refused to participate in the program for various reasons, such as lack of interest, too much paperwork, or intimidation by the quiz-like set-up. Chain pharmacies were associated with the least improvement in post-intervention scores. Although the potential variables associated with this result need further exploration, this result suggests a possible need to tailor intervention implementation by location in future scaled-up initiatives. Feedback from pharmacy interns suggested that omitting the PKQ would increase engagement of participants in the program. After receipt of feedback from the first 6-week rotation of interns, additional tips were provided to interns to increase patient participation. One tip was to include a return-reply envelope for patients who declined to complete the post-intervention PKQ.

Patient knowledge of NSAID kidney-safety issues improved as a result of this community pharmacy–based education program. Pharmacists are easily accessible health care providers and can augment primary care office- based education programs. Based on the observed need for patient education on NSAID avoidance, we augmented our core curriculum on the topic of medications and renal hemodynamics for doctor of pharmacy candidates. The success of this project led to collaboration with the National Kidney Disease Education Program to develop an innovative, interactive continuing education program on NSAID avoidance for pharmacists and other health care providers. The program is available nationally at www.nkdep.nih.gov.
